# A Microfluidic Platform for Single Cell Fluorometric Granzyme B Profiling

**DOI:** 10.7150/thno.37728

**Published:** 2020-01-01

**Authors:** Jonathan C. Briones, Wilfred V. Espulgar, Shohei Koyama, Hiroyuki Yoshikawa, JeongHoon Park, Yujiro Naito, Atsushi Kumanogoh, Eiichi Tamiya, Hyota Takamatsu, Masato Saito

**Affiliations:** 1 Graduate School of Engineering, Osaka University, Suita, Osaka, 565-0871, JAPAN.; 2 Graduate School of Medicine, Osaka University, Suita, Osaka, 565-0871, JAPAN.; 3 AIST PhotoBIO-OIL, Osaka University, Suita, Osaka, 565-0871, JAPAN.

**Keywords:** single cell, granzyme b profiling, microfluidics, immunotherapy

## Abstract

Granzyme B (GrB) is an essential cytotoxic effector in cancer immunotherapy as it can be a potential biomarker to predict the efficacy of immunotherapies including checkpoint inhibitors. Monitoring the Granzyme B activity in cells would help determine a patient's clinical response to treatment and lead to better treatment strategies by preventing administration of ineffective therapies and avoid adverse events resulting in a delay in subsequent treatment.

**Methods**: A microfluidic device with hydrodynamic traps and pneumatic valving system was fabricated using photo and soft lithography. Single cell Granzyme B (GrB) activity was detected and measured fluorometrically using a commercial assay kit with a peptide substrate containing GrB recognition sequence (Ac-IEPD-AFC) and AFC (7-Amino-4-trifluoromethylcoumarin) label. Fluorescence was observed and measured using a confocal microscope with CSU-W1 scanner unit and CCD camera as well as an inverted microscope with photodetector. Model cells (NK-92, GrB-transduced Jurkat, and THP1 cells) and human PBMCs from healthy donor and lung cancer patients including an anti-PD-1 antibody treated patient were profiled of its GrB activity as proof of concept.

**Results**: GrB expression from the model cells was found to be markedly different. NK-92 cells were found to have higher GrB activity than the GrB-transduced Jurkat cells. THP-1 was found to have relatively no significant activity. A marked increase in GrB expression was also observed in anti-PD-1 treated lung cancer patient sample in comparison to PBMC from a healthy donor. TCR+ Ig-G4+ PBMC cells were found to have high activity which signifies a clear response to PD-1 blockade.

**Conclusion**: As proof of concept, we have shown the capability of a microfluidic platform to measure GrB production through a single cell enzymatic activity assay. Our platform might be a promising tool for evaluating the sensitivity of immunotherapies and identifying specific T cell subset responsible for the anti-tumor response.

## Introduction

Granzyme B (GrB) is a serine protease that is responsible for the rapid induction of caspase-dependent apoptosis 1-2. Together with a pore-forming protein called perforin and a number of other associated proteases in a cytotoxic granule, these granular contents are secreted by cytotoxic lymphocytes, such as CD8+ T cells, γδ T cells, natural killer (NK) cells and NK T cells, as primary tumor killing mechanism upon target cell recognition, 1,3-5. The expression of tumoral GrB represents the incorporation of cytotoxic T cell tumor localization and tumoricidal activity as well as immune-mediated cell killing enhancements or suppression 3. Thus, monitoring the GrB activity in cells would help determine a patient's response to treatment 3, 5-6.

Discriminating the responding from the non-responding patients would lead to better treatment strategies as this can prevent the administration of ineffective therapies and avoid adverse events.

Most reports on GrB quantification make use of bulk samples in approaches like immunehistochemistry, real-time PCR, and western blot analysis 3, 7-8. Using similar methods, PET imaging with GrB-specific PET imaging probe has been reported to successfully measure tumoral GrB expression between immunotherapy responders and non-responders 3. Immunoassay platform such as enzyme-linked immunosorbent spot (ELISpot) and multiplex platforms (Luminex® and EllaTM] have also been explored to measure biomarker activity 9-10. On the other hand, Flow cytometry analysis (FACS) has been used to ORFV-activated NK cells to determine IFNγ and GrB expression but it did not explore the variability of expression in single cell level 11. Conventional bulk analyses are not enough to extract information on cells that can be used for development and personalization of treatments. Specifically, cell subsets with GrB overexpression that is responsible for anti-tumor response cannot be shown in the bulk approach as this gives only a stochastic average 12-15. This measurement approach ignores the statistical nature of many cellular events and masks molecular details such as variations in the protease expressions of the different immune cells 15-18. Thus, a single cell approach is needed.

There are several single cell technologies that are available to study individual behaviour of cells and characterize the dynamics of immune response under varied conditions. One of these is Fluorescence-activated cell sorting or FACS. A limitation of FACS, aside from high cost, is not being able to track target single cells over time 15, 19. On-chip incubation to detect and measure cell secreted proteins would also be difficult using the said platform 20. GrB detection by FACS would also require cells to be fixed, not live cell 20, 21. Other techniques for single cell analysis like droplet microfluidics may have the capability to do high throughput screening and analysis at several time points. However, this approach is limited by the low cell encapsulation rate, large populations of empty droplets, reducing the downstream readout efficiency. Jetting microfluidics with size sorting capability has resolved this concern by collecting only the droplets containing cells. This technique has been used for single-cell protease detection 22 Nevertheless, this is difficult to use for protocols that require the exchange of aqueous environment like washing steps or peptide substrate exposure in immunoassays 12. Optofluidics and optical traps require expensive lasers and accurate alignment while microwells and hydrodynamic trapping in microfluidic devices necessitates microfabrication for device production 23.

The lack of reports on single cell analysis is mainly due to the absence of a simple assay platform for single-cell measurement. Therefore, single cell level of monitoring was sought to investigate the variability in single cell GrB production as well as identify T-cell subsets in the profile that shows high protease expression. The required platform for this, aside from being low cost, must be able to determine the magnitude of GrB expression of each immune cell and show the diversity of the protease expression within the cell population. A platform that is capable of measuring at protein level and collecting of the GrB-highly producing cell is advantageous as compared to single cell RNA sequencing.

At present, biosensing platforms using fluorescence measurement have become a convention for detecting and quantifying biomolecules like DNAs, miRNAs, proteins, and enzymes for both bulk and single cell analysis. Label free assays for other apoptotic proteases like caspase-3 and caspase-7 have been developed to homogenously detect proteases* in vitro* and in complex cell lysate. Quantification was done by measuring the change in fluorescence as a result of the cleavage of a modular peptide by the said protease and the removal of a di-cysteine motif from peptide, which abrogates the bipartite tetracysteine display 24. Single-molecule detection technology have recently been reported using Förster resonance energy transfer (FRET) technology to count Cy5 bursts, which indicate the presence of target molecule 25. FRET modified substrates have also been developed to accommodate different fluorescent pairs with distinct excitation and emission wavelengths in order to obtain multiple signals of enzymes from single-cell encapsulated droplets and characterize protease activity profiles at single cell resolution 26. Being a common tool in clinical and biological labs and familiarity of most users, fluorescence based detection was sought after in GrB measurement in this study.

In this work, we fabricated a high throughput single cell screening microfluidic platform that can do compartmentalization and on-demand media exchange for repeated measurements. The current design of the microfluidic chip was inspired by the work of Armbrecht and Dittrich for parallel analysis and monitoring of a large number of isolated cells 12. Pneumatic valves were integrated into the chip to enable the rapid and repeated fluid exchange. Cells were mechanically captured in hydrodynamic traps and isolated in separate microchambers of about 70 pL in volume with the actuation of the pneumatic valves. A fluorometric activity assay was performed to measure GrB expression through its cleaving of a peptide substrate and release of AFC label. The expressed proteases from human immune cell lines (NK-92, GrB transduced Jurkat, and THP-1 cells) were compared using the single cell approach and the bulk approach. The platform was also applied to human PBMC samples from healthy donors and lung cancer patients including anti-PD-1 antibody-treated patients. Cell surface marker staining was performed to distinguish specific cell populations producing GrB. Aside from GrB, immune cell expression of the other members of the Granzyme family can be investigated of their activities in immune response as well as collection of the cell of interest for further analysis as a possible extension of the study.

## Methods

### Microfluidic chip fabrication

The microfluidic chip consists of two PDMS layers, one is a thin “flow” layer that contains an array of hydrodynamic traps as well as other microstructures that serve as filters, bubble traps, and pillar flow guides. Here, cell samples and reagents were introduced through an inlet and made to flow through the channel with the use of a syringe pump (YSP-201, YMC Co. Ltd). The second is a thicker “control” layer with pneumatic valves that can be actuated with positive pressure to create a sealed chamber of about 70 pL volume. To create the device, two master moulds were first fabricated using optical photolithography. The structures on the control layer were enlarged by 1.5% to account for shrinkage.

For the flow layer, SU8 3010 and 3025 photoresists (MicroChem, Massachusetts, USA) were spin coated at different steps on a 4”silicon wafer to create structures of varying heights of 15 µm and 25 µm. The resist was exposed to UV light source (20,000 mJ cm^-2^ at 405 nm) using a maskless exposure apparatus (Nano System Solutions DL-1000). After development (SU-8 Developer, MicroChem, Massachusetts, USA), hard baking, and installation of metal posts, the master forms were silanized with 1H,1H,2H,2H-perfluorodecyltrichlorosilane (Wako Pure Chemical Industries, Ltd.) for ease of release of PDMS from the master mould. The master mould for the control layer was fabricated with the same procedure using SU8 3025 to create 20 µm and 50 µm structures.

For chip fabrication, the PDMS oligomer and curing agent (Sylgard 184 silicone elastomer kit, Dow Corning, USA) were mixed at a ratio of 10: 1 and 5:1 for the flow layer and control layer, respectively. Afterwards, the mixture was degassed for 15 min, poured onto the control layer master, and soft baked at 80 °C for 15 minutes. Silicone tubes (1mm inner / 3mm outer diameter) of 10 mm length were attached to the metal posts that serve as inlets prior to baking. For the flow layer, about 3 mL of the mixture was spin-coated at 3000 rpm for 60s onto its corresponding mould to produce about 30 µm thick PDMS layer. This was then baked at 80 °C for 10 min.

**Figure 1:**
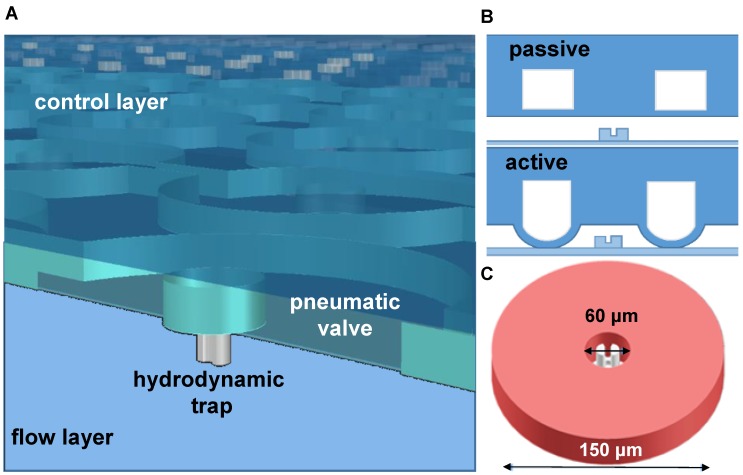
An illustration of the two layers of the microfluidic device. A) A 3D rendering of the microchambers with the pneumatic valve on top and hydrodynamic trap at the bottom. B) Actuation of the valve deflects a membrane to create a sealed chamber. C) A mechanical trap located at the center of the chamber with about 30 µm diameter.

**Figure 2:**
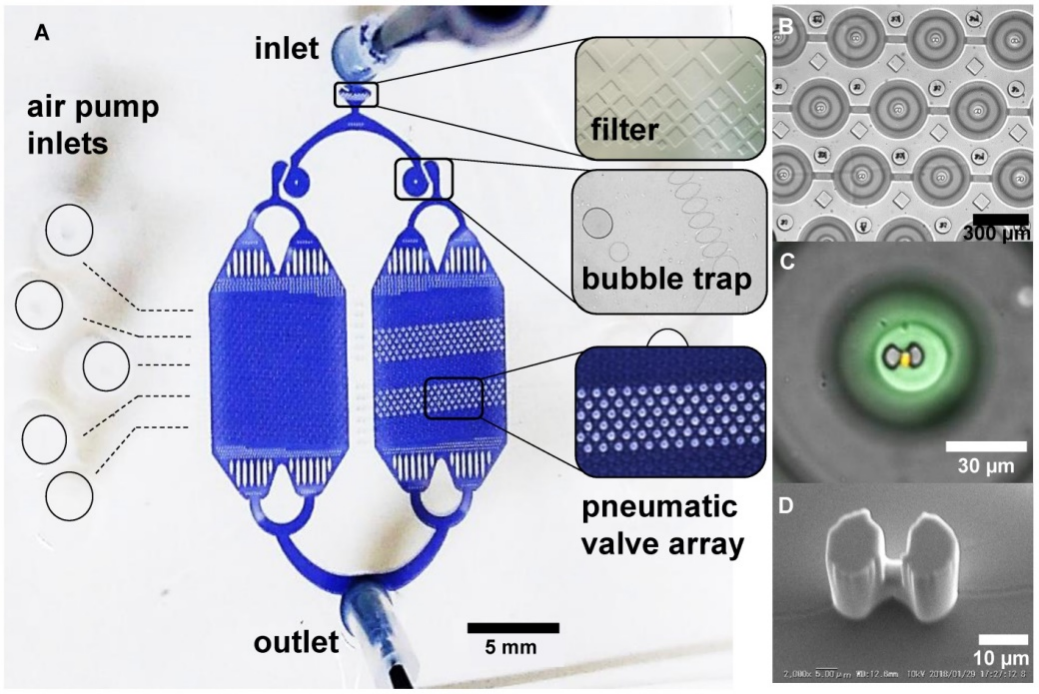
A microfluidic platform for single cell compartmentalization. A) An image of the chip with (inset) portions of actuated chambers. B) An array of actuated chambers C) A magnified view of a sealed chamber with a trapped model cell (yellow) and fluorescent media (green). D) SEM micrograph of the hydrodynamic trap at the center of the micro chamber.

After the control layer was cured and peeled off from the mould, it was cut to desired size and then aligned on top of the spin-coated flow mould. The remaining 5:1 PDMS mixture was poured around the aligned layers and then hard baked for 2 hours at 80 °C, resulting to the bonding of both layers. The PDMS chip was finally bonded to a cleaned microscopy coverslip after exposure to oxygen plasma for 20 s in a Plasma Dry Cleaner (PDC210, Yamato) at maximum power (75 W). The detailed description of the master mold fabrication and chip fabrication as well the microfluidic chip design specification are available in the supplementary papers (**Figure S1 and S2**). The upper PDMS layer contains donut shaped valves that are actuated by filling with air using an air pump (DA-60S, ULVAC). **Figure 1** illustrates the actuation of the valve and the resulting microchamber. The actuation test to determine the valve's working pressure is shown in **Figure S3**. **Figure 2A** shows a picture of the microfluidic chip filled with a colored liquid for visualization. A magnified view of its parts, particularly the microchambers (**Figure 2B-C**) and the hydrodynamic trap structure (**Figure 2D**) is shown.

### Cell culturing, viability and identification

As proof of concept, model cells were initially used in the conduct of the assay experiment. These include Natural killer NK-92 Cell, GrB-transduced Jurkat T-lymphocyte cell, and THP1 monocyte cells. The model cells were provided by the collaborators from Osaka University Hospital and were expanded from a cryopreserved master cell bank. The NK-92 cells were maintained in alpha medium supplemented with 2 mM L-glutamine, 0.2 mM I-inositol, 20 mM folic acid, 10-4 M 2-mercaptoethanol, 12.5% fetal bovine serum (FBS), and 12.5% horse serum in the presence of 500 IU/ml human interleukin-2 (IL-2). The Jurkat and THP1 cells were maintained in RPMI 1640 medium with 10% FBS and 1% penicillin. The same collaborators also provided peripheral blood mononuclear cells (PBMC), from healthy donors and PD-1 antibody treated lung cancer patients, which were revived and stored in the same media as Jurkat and THP1 cells. All handling and experiments on cell samples were performed in accordance with the protocols of the Research Safety Committee of Osaka University.

GrB-overexpressing Jurkat cells were generated by lentiviral transduction of expression plasmid. Briefly, a CDS of GrB was cloned from a commercially available GrB plasmid (Origene, RC206495) and inserted into lentiviral vector CSII-EF-MCS-IRES2-Venus (RIKEN, Dr. Miyoshi's lab). Jurkat cells were transduced with lentiviral GrB plasmids by spinfection. Successful plasmid transduction was confirmed by the expression of venus and monoclonal cells and were isolated by manual cell picking under microscopy.

For visualization, Propidium iodide (PI, Sigma-Aldrich, USA) staining of cells was performed. PI is a red fluorescent indicator for dead cells that act by intercalating with cellular DNA 27. The staining with PI was done to evaluate the viability of the cells in the chip. A 1:2000 of stock solution, 2 mg/ml Propidium Iodide in PBS (phosphate buffered saline, Sigma-Aldrich, USA), was prepared. PI was then added to the media perfused through the microfluidic device. Fluorescent images were taken using a confocal microscope (IX83, Olympus) at 535/617 nm excitation/emission.

To identify the composition of PBMC samples, cell surface marker staining was performed. Anti-CD56 human antibody (Alexa Fluor 405, R&D Systems, USA and Alexa fluor 647, Biolegend, USA) was used to identify natural killer cells. CD56 is the archetypal phenotypic marker most stringently associated to natural killer cells but can also be expressed on the surface of some T cells 28. The Anti-human TCR αβ (Alexa Fluor 647, Biolegend, USA) which reacts with a monomorphic determinant of the α/β T cell receptor was used to determine T-cells. Co-expression of TCR αβ and CD56 receptors in NK T cells can result to double positive staining (CD56+ TCR+). Monocytes, meanwhile, were identified with Anti-CD14 human antibody (Alexa Fluor 405, R&D systems, USA). Additionally, patient PBMC were subjected to IgG4 immunostaining to identify specific T cell subset responsible for anti-tumor response.

### Device operation

**Figure 3:**
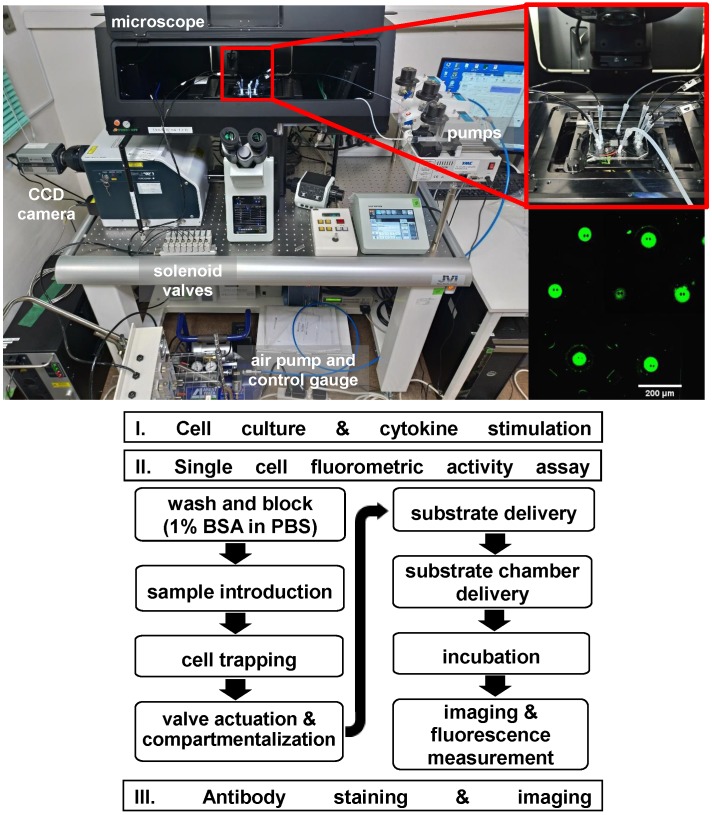
Above: The single cell assay system uses a microfluidic device connected to fluid and air pumps. An inverted microscope with a 405 nm exciting laser and CCD camera was used in detecting fluorescence. Inset, a magnified view of the microfluidic chip and an image of the chambers with fluorescence signal. Below: Flowchart of the single cell fluorometric activity assay protocol.

Fluorometric Granzyme B activity assay and staining were performed in the microfluidic device. A commercial assay kit, PromoKine Fluorometric Granzyme B Activity kit (Promocell GmbH, Heidelberg, Germany) was used. Prior to assay experiment, all cells were maintained in medium containing with IL-2 (500 IU/ml) in an incubator at 37°C and 5% CO_2_ for 12 hours. To reduce cell adhesion on the channel walls, the chip was blocked with 1% BSA in PBS solution (bovine serum albumin, Sigma-Aldrich, USA) for 30 mins. Using a syringe pump, the chip was washed with PBS for 10 min at a flow rate of 10 µL min^-1^. A 50 µL cell suspension (10^6^ cells mL^-1^) was then flushed into the chip at a flow rate of 10 µL min^-1^ to be captured in the hydrodynamic traps. With the same flow rate, excess cells were flushed out by rinsing the chip with 50 µL of buffer medium. Afterwards, the pneumatic valves were pressurized with 0.07 MPa to seal the microchambers and isolate each trapped cell. After flushing the chip with Granzyme B recognition substrate (Ac-IEPD-AFC) in buffer, the pneumatic valves were briefly opened and closed to allow the substrate to enter the chamber and interact with the cell. Thirty minutes incubation is then performed. The non-fluorescent substrate is hydrolysed by the expressed Granzyme B in an enzyme-catalyzed reaction which resulted in the release of AFC proportional to the enzymatic activity present. Both an inverted microscope (IX70, Olympus) with photodetector (Femtowatt Silicon Photoreceiver, New Focus Sigma Koki), λ = 405 nm excitation laser (LAVIOS Laser Module, excitation power = 1.7 mW), and inverted microscope (IX83, Olympus) with Confocal scanner unit (CSU-W1, Yokogawa), CCD camera (Zyla sCMOS, ANDOR Tech) were used to observe and measure the fluorescence in the microchambers. However, using the former took longer time to measure more than a hundred chambers, thus imaging using the confocal microscope was performed. On the other hand, it shows the flexibility of the platform to be used in different microscope systems. The acquired data was then analyzed and plot using ImageJ image analysis software. **Figure 3** shows the experimental set-up and the flowchart of the experiment protocol. To compare with conventional methods, the GrB activity assay was also performed on a 96 well plate for bulk samples. The standard curves obtained using the microfluidic device for single cell measurement and a 96-well plate for bulk sample measurement can be found in the supplementary pages (**Figure S4**).

Following the assay kit protocol, different concentrations of the AFC standard in granzyme B assay buffer was prepared (ex. 0, 10, 20, 50, 100, 150, 200 and 250 pmol). Fluorescence measurement was taken using a confocal microscope and the standard curve was generated. The fluorescence measurement used was based from the maximum observable intensity after a certain incubation time. One unit of Granzyme B is the amount of enzyme that hydrolyzes 1 pmol of Ac-IEPD-AFC per min. The fluorescence intensity measurements from the chambers were interpolated from the standard curve to acquire information on GrB expression.

Prior to the assay experiment, the chip was tested for its ability to trap and isolate single cell. A 60 µL of cell suspension (106 cells mL-1) was introduced into the chip and made to flow at different flow rates of 5, 10, 15, 20, and 30 µL min-1. Trapped cells were imaged using an inverted microscope and visually inspected for single, double, and more than 2 captured cells. These trapped cells were manually counted. Only the chambers with traps containing one cell were used analyzed.

## Results and Discussion

Sample cells that are introduced and made to flow in the flow channel follow a streamline and is captured when it passes through the trap gap. The gap size is smaller than the cell diameter and thus allow cells to be mechanically stopped and immobilized tightly in place. Pillars serving as flow guides are placed to coerce the streamlines to pass through the trap gap. Velocity distribution creates different pressure from the main channel to the trap gap. The flow velocity in the trap gap is faster than the main channel and this allows a particle to be driven into a trap by hydrodynamic forces when the trap is empty.

**Figure 4:**
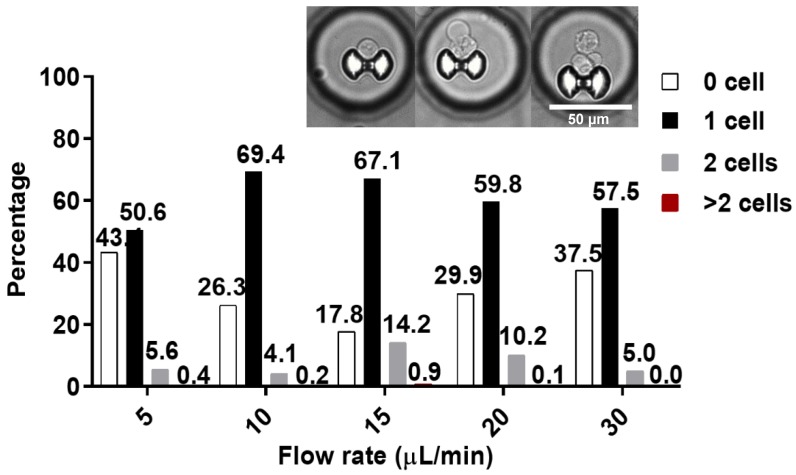
Percentage of trap occupancy at different flow rates. The percentage of traps with single capture is shown as a black solid bar, double capture as a gray solid bar, and traps with more than 2 cells is shown as solid red bar. Only traps with single cell were analyzed. Inset, representative images of the captured cells.

More than 500 microchambers, on average, contains a single trapped cell. At 5 µL min^-1^, about 50.6% of the traps were occupied by single cell and 5.6% by two cells. At 10 µL min^-1^, 69.4% and 4.1% of the traps were occupied by one cell and two cells, respectively. At 15 µL min^-1^, 67.1 % for single and 14.2% double occupancies were reached. At 20 µL min^-1^, 59.8% and 10.2%; while at 30 µL min^-1^, 57.5% and 5% single cell and double cell occupancies were reached, respectively. Less than 1% of the traps were occupied by 3 or more cells for all flow rates (**Figure 4**). Traps with more than 1 cell were excluded from the assay data evaluation process. The flowrate of 10-µL min^-1^ was later on used for cell trapping in the assay experiment. Additional details on the trapping test can be found in the supplementary papers (**Figure S5**). The hydrodynamic trap was designed in such that its height and opening are close to the diameter of a single cell, to prevent multi-cell stacking. This is reflected by the low percentage of trap occupancy for more than 1 cell. Cells tend to squeeze through trap more often at high flow rate like 10 µL min^-1^, but tends to miss the target or bounce off the trap and result to cell loss at even higher flow rate.

**Figure 5:**
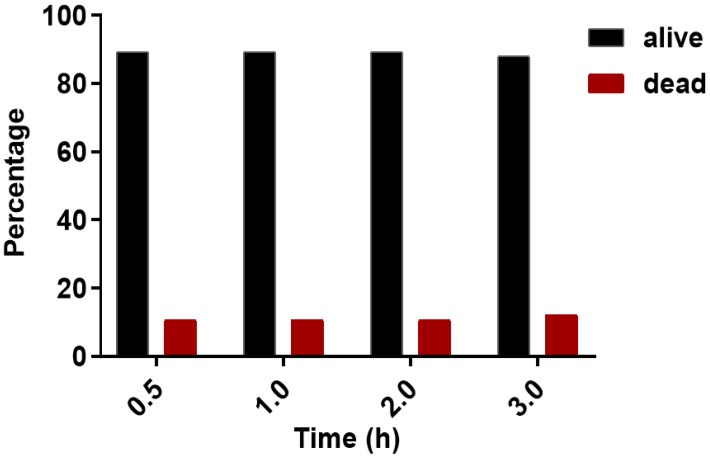
Percentage of alive and dead cells at different observation times. Propidium iodide (PI) staining of cells was done to determine dead cells. The black and red bar represents alive and dead cells, respectively.

Propidium iodide (PI) staining was performed to evaluate on-chip viability of cells. PI was added to the media perfused through the microfluidic device. The cell in each chamber, exposed to PI, was observed before and after valve actuation. The valves were left actuated for three (3) hours to observe its effect on cell state. Before actuation 89.4% of cells were found to be alive and 10.6% were dead (**Figure 5**). One and two hours after continuous actuation, the same number of alive and dead cells were found. After 3 hours of actuation, 88% of the cells were found to be alive and 12% were dead (**Figure 5**). The chip operation was, therefore, observed to have no significant effect on cell state during the experiment period.

**Figure 6:**
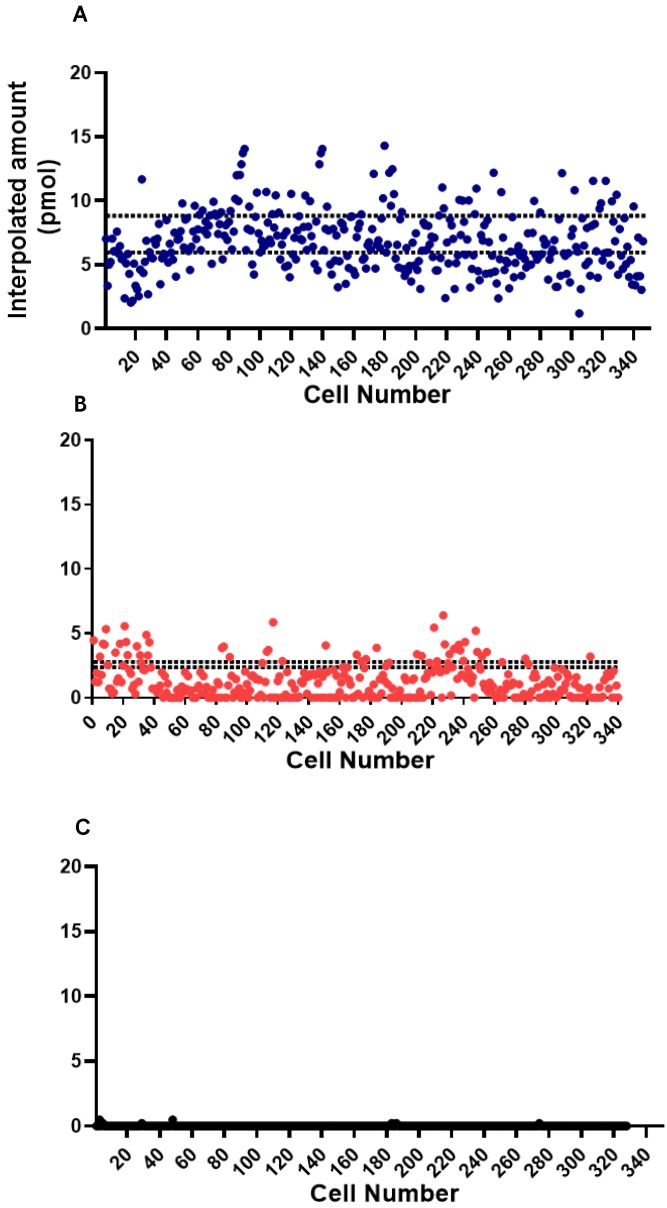
Single cell fluorometric Granzyme B activity assay for (A) NK-92 (blue square), (B) Jurkat (red circle), and (C) THP1 (black triangle) model cells. The dashed lines indicate the maximum and minimum measurements for bulk sample (N=5).

In the conduct of the assay experiment, single cell approach was done using the microfluidic device. The model cells, GrB-transduced Jurkat T Lymphocyte cell, NK-92 natural killer cell (positive control), and THP1 monocyte cell (negative control), were used to test the ability of the device to detect and measure the fluorescence from the GrB activity. The single cell assay experiment was conducted on separate microfluidic chips for each cell type. The background was taken from an actuated chamber without substrate and later on subtracted from assay measurements. The measurements were interpolated from the standard curve to acquire information on GrB expression. As a proof-of-concept, around 350 chambers with trapped single cell were used in the observation and measurement of fluorescence from the GrB activity of each model cells. There was a marked difference in the GrB expressions of each cell type. A variation in GrB activities of each cell was recorded with NK-92 samples having a higher GrB activity than the other two types of cells (**Figure 6**). The expression of observed NK-92 cells in the microchambers was interpolated to reach a max. of 14.3 pmol (**Figure 6A**) while the Jurkat cell reached 6.4 pmol of expressed GrB (**Figure 6B**). THP-1 was found to have near zero activity (**Figure 6C**).

To compare with the conventional approach, the assay experiment as also conducted on bulk samples. Fifty microliters (10^6^ cells/ml) cell suspension of the model cells were pipetted in a 96-well plate together with a 50 µl reaction mix (5 µl GrB recognition substrate and 45 µl buffer). The fluorescence of the cleaved AFC from each sample was observed and measured after 30 minutes of incubation. The measurements were also interpolated from its standard curve to determine GrB concentration. Background was taken from a well containing cells and buffer but without the peptide substrate. Background measurement was subtracted from the sample measurements. GrB expressions from these cells were noticeably different. Comparing the model cells, NK-92 cells were found to have higher GrB activity than the Jurkat cells as represented by the dashed lines in **Figure 6** for each cell type, N=5. After the incubation time, NK-92 cell sample was interpolated to have a mean GrB concentration of 7.4 pmol (max. of 8.8 pmol; min. 6 pmol). Meanwhile, the jurkat cell sample has a mean expression of 2.6 pmol (max. of 2.8 pmol; min. of 2.4 pmol). THP-1 was found to have relatively no significant activity.

**Figure 7:**
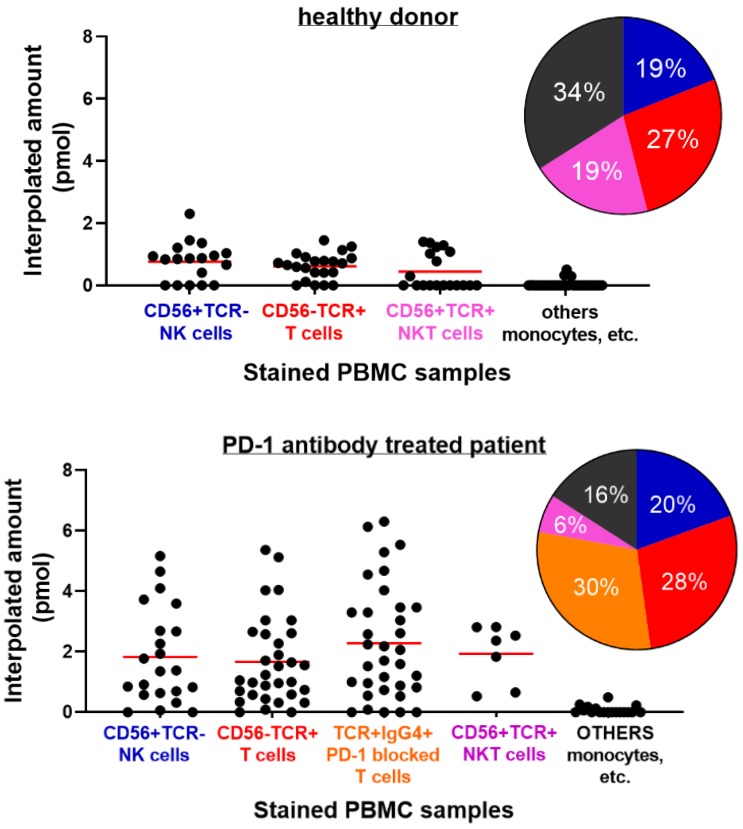
Single Cell Fluorometric Granzyme B Activity of PBMC samples from (top) healthy donor and (bottom) PD-1 antibody treated lung cancer patient. Antibody staining was done to the PBMC sample to distinguish the types of cells present. Each cell type is grouped according to their corresponding markers. Solid line indicates mean value for the group. Pie chart refers to the percentages of each type of (stained) cells in the PBMC sample that were analyzed for Granzyme activity using the microfluidic device. Colors follow cell type assignment in the data plot (P<0.0001, healthy vs. patient).

Next, PBMC samples were used in the single cell assay experiment. One of the sets of samples came from healthy donors and the other set are from lung cancer patients treated with anti-PD-1 antibody checkpoint inhibitors. Again, as proof-of-concept, around 110 chambers with trapped single cells were used in the observation of GrB activity from each set. Comparison of the single cell fluorometric enzyme activity of the PBMC cells from healthy donor and patient was sought. The cell samples later on went through cell surface marker staining to identify some of its composition. GrB activities from cells identified with CD56+ TCR-, CD56- TCR+, CD56+ TCR+ double positive markers, and unstained cells were grouped as shown in **Figure 7** and**Figure S6**. IgG4 immunostaining was also performed to the PBMC from a patient in order to determine specific T cell subset responsible for anti-tumor response. IgG4+ TCR+ stained cells are therapeutic antibody bound T cells 29. The percent composition of each type of immunostained cell in the PBMC samples that were analyzed for the GrB activity using the microfluidic device is shown as a pie chart in **Figure 7**.

Cells stained with anti-CD56+ TCR-, believed to be NK cells from PBMC of a healthy donor, were found to reach about 2.3 pmol of GrB activity. While both cells stained with anti-TCR αβ (CD56- TCR+), believed to be T cell, and double positive stain (CD56+ TCR+), possibly T cell or NK-T cell, reached an interpolated value of about 1.45 pmol and 1.41 pmol GrB activity, respectively. Unstained cells, believe to include monocyte cell types, were found to have near zero activity. On the other hand, GrB activity of PBMC cells from patients was found to be higher than the result from the healthy donor. Cells with CD56+ TCR- and CD56- TCR+ stains were recorded to reach interpolated values of 5.2 pmol and 5.4 pmol, respectively. Meanwhile, T cells that are IgG4 positive (Ig-G4+ TCR+) reached 6.3 pmol interpolated value, about a unit higher than CD56- TCR+ and CD56+ TCR- cells. Unstained cells were still found to have near zero activity. The high GrB activity of the Ig-G4+ TCR+ cells in the patient PBMC signifies a response to PD-1 blockade and its antibody treatment. The increase in the expression or potency of the anti-PD-1 bound T cells as compared to the unbound or nonmodified subset suggests the effectiveness of the therapy and demonstrates the platform's potential in the said therapeutic strategy. Identification of the T cells bound with IgG4 and its GrB activity provides information on the response of the tumor to the treatment. Using the Mann-Whitney test or Wilcoxon rank sum test to compare the GrB activity of the healthy and patient PBMC samples, a P value of <0.0001 was obtained suggesting a significant difference between the groups.

The use of the microfluidic platform in the single cell GrB activity assay has shown variation in the GrB activity between the healthy donor's PBMC and that of the patient's. Increase in GrB expression, as well as perforin, by peripheral blood CD8+ T lymphocytes or other lymphocytes have been seen in chronic diseases 20 Recent studies have given focus on the utility of GrB in the blood as a biomarker of immune activation in humans with a variety of disorders 30. The elevated GrB production that was observed in peripheral blood mononuclear cells isolated from patients could provide information about the disease and thus shows the platform's potential utility for diagnosis. Although it is assumed that the elevated GrB levels are due to increased expression by CTL activation; the contribution from other immune cells is lacking in reports 31.

In this work, we demonstrated the capability of the microfluidic platform to determine single cell GrB production through a fluorometric enzyme activity assay. Our platform has the potential for evaluating the sensitivity of immunotherapies or predicting the response of the tumors to these therapies. Our approach is very simple, requiring only a small amount of cell samples and reagents, making it applicable to clinical setting.

The single cell experiment showed variation is each cell activity which is not observed in the bulk approach. This variability of cell response gives a profile of information, which is meaningful in designing and personalization of treatments. This potentially can provide an early and quantitative verification of successful therapeutic development. By comparing the protease expression of the immune cells, specific T cell subset responsible for the anti-tumor response can be identified. These cells with overexpression of GrB has been an interest especially in immune checkpoint inhibitors immunotherapy and adaptive cell transfer therapy 31-32.

In principle, the engagement of PD-1 through its ligand, PD-L1 (programmed death ligand), negatively regulates T cell mediated immune responses and serves as a mechanism for tumors to evade said response. Activated T-cells express PD-1 receptors on its surface and blocking these with antibodies, i.e. IgG4 antibody, reverses the processes thereby enhancing antitumor immune activity 33. Identification of IgG4+ TCR+ cells in the patient's blood with overexpression of GrB is significant in the investigation on therapy response and treatment effectiveness. Retrieval of these cells for further study would provide information into its cytotoxic property and mechanism. On the other hand, performing mRNA sequencing to identify the unique genes responsible for the overexpression of GrB would be important in the development of new strategies for cancer immunotherapy. Such a platform would allow for repeated noninvasive and systemic interrogation of the tumoral response to cancer immunotherapy and provide insight into a major biochemical mechanism responsible for immune-mediated cell death. Aside from Granzyme B, the activity of other proteases such as Granzyme A, K, H, and M can also be explored in the said platform to better understand their activities and possible mode of action immune response.

## Conclusions

As proof of concept, we have shown the capability of a microfluidic platform to measure GrB production through a single-cell enzymatic activity assay. The study of single cell expression of granzymes is seen important in the medical field as a promising tool in evaluating immunotherapy response. GrB levels in the blood can be profiled to identify specific T-cell subset responsible for anti-tumor response. Going forward, the next challenge is the retrieval of the identified lymphocyte cells in the patient PBMC with GrB overexpression. Further analysis can be done to find out the factors contributing to its overexpression and the mechanism for its cytotoxicity. This may open up new perspectives on cancer immunotherapy research.

## Supplementary Material

Supplementary figures.

## Abbreviations

AFC: 7-Amino-4-trifluoromethylcoumarin
BSA: bovine serum albumin
GrB: Granzyme B
CTL: cytotoxic T lymphocyte
FACS: fluorescence-activated cell sorting
FBS: fetal bovine serum
NK: natural killer cell
PBMC: peripheral blood mononuclear cell
PBS: phosphate buffered saline
PD-1: Programmed cell death protein 1
PD-L1: programmed death ligand
PI: propidium iodide
