# Supplementary material for: A Microfluidic Platform for Single Cell Fluorometric Granzyme B Profiling
Source: Theranostics. 2020 Jan 1;10(1):123–32. doi: 10.7150/thno.37728 (PMC6929635; doi:10.7150/thno.37728)
Supplement: Supplementary file 1 — Supplementary figures. [file thnov10p0123s1.pdf]

Supporting Information for

A microfluidic Platform for Single Cell Fluorometric Granzyme B Profiling

Jonathan C. Briones [1†], Wilfred V. Espulgar [1†], Shohei Koyama [2], Hiroyuki Yoshikawa [1], JeongHoon Park [2], Yujiro Naito[2], Atsushi Kumanogoh [2], Eiichi Tamiya [1,3*], Hyota Takamatsu [2], and Masato Saito [1,3]

1. Graduate School of Engineering, Osaka University, Suita, Osaka, 565-0871, JAPAN.

2. Graduate School of Medicine, Osaka University, Suita, Osaka, 565-0871, JAPAN.

3. AIST PhotoBIO-OIL, Osaka University, Suita, Osaka, 565-0871, JAPAN.

† These authors contributed equally to this work.
* Authors to whom the correspondences should be addressed: E. Tamiya (tamiya@ap.eng.osaka-u.ac.jp) and M. Saito (saitomasato@ap.eng.osaka-u.ac.jp).

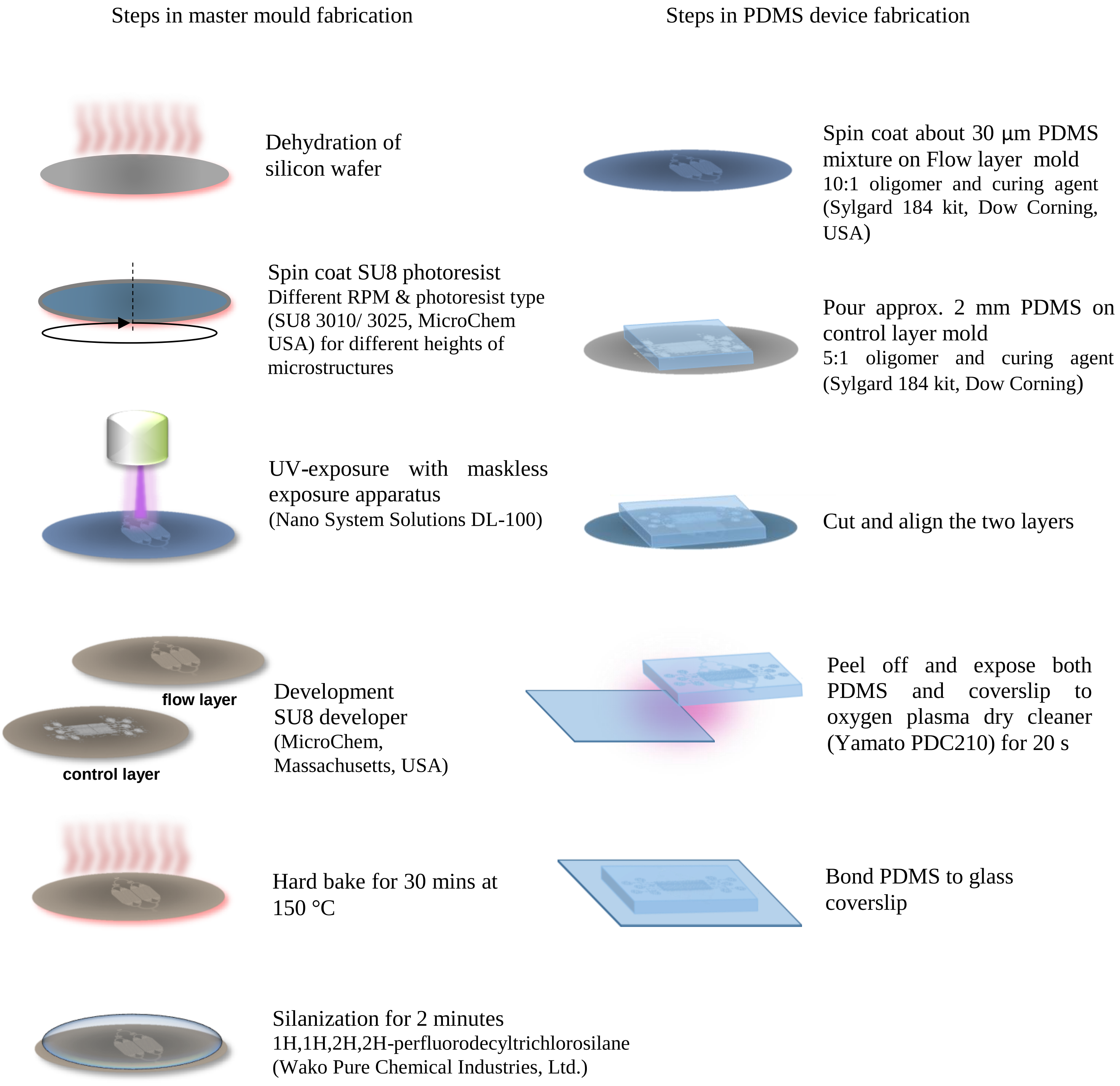

Figure S1. Fabrication of the master mold by soft-lithography of SU-8 (3010 and 3025) on 4” Si-wafers and of microfluidic PDMS chip from the silicon master molds.

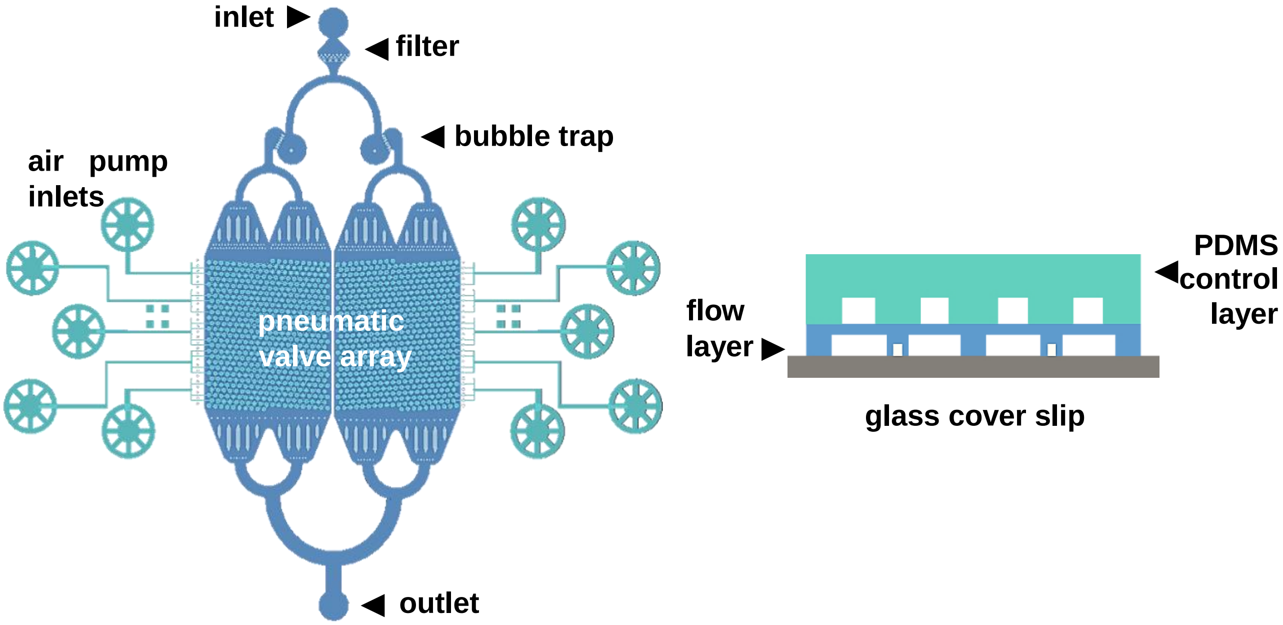

Left, combined CAD design of the control (cyan) and flow layer (blue) moulds.
Right, representation of the PDMS chip assembly.

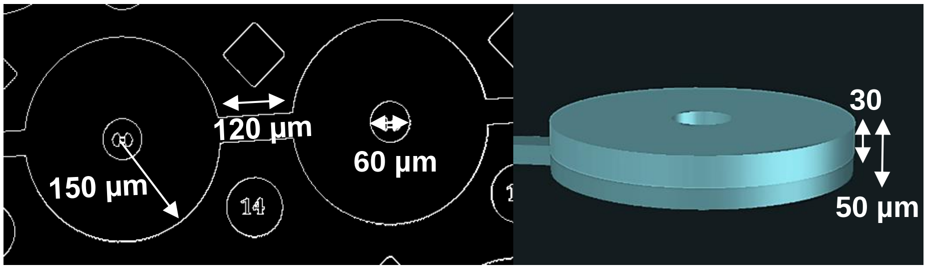

pneumatic valve dimensions

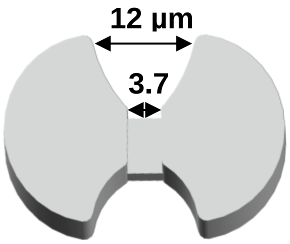

hydrodynamic trap design

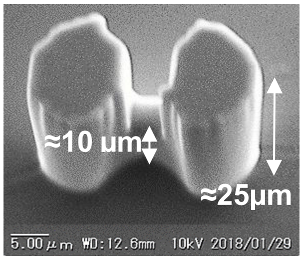

resulting PDMS trap

Figure S2. Microfluidic chip design and specifications of its main parts. The PDMS device has two layers, a flow layer at the bottom and a control layer on top. The flow layer has microstructures which includes a filter, bubble trap, flow guide pillars, and hydrodynamic traps. The control layer is made up of pneumatic valves, which are cylinder shaped. The actuation of this valves creates a microchamber with the mechanical trap at the center.

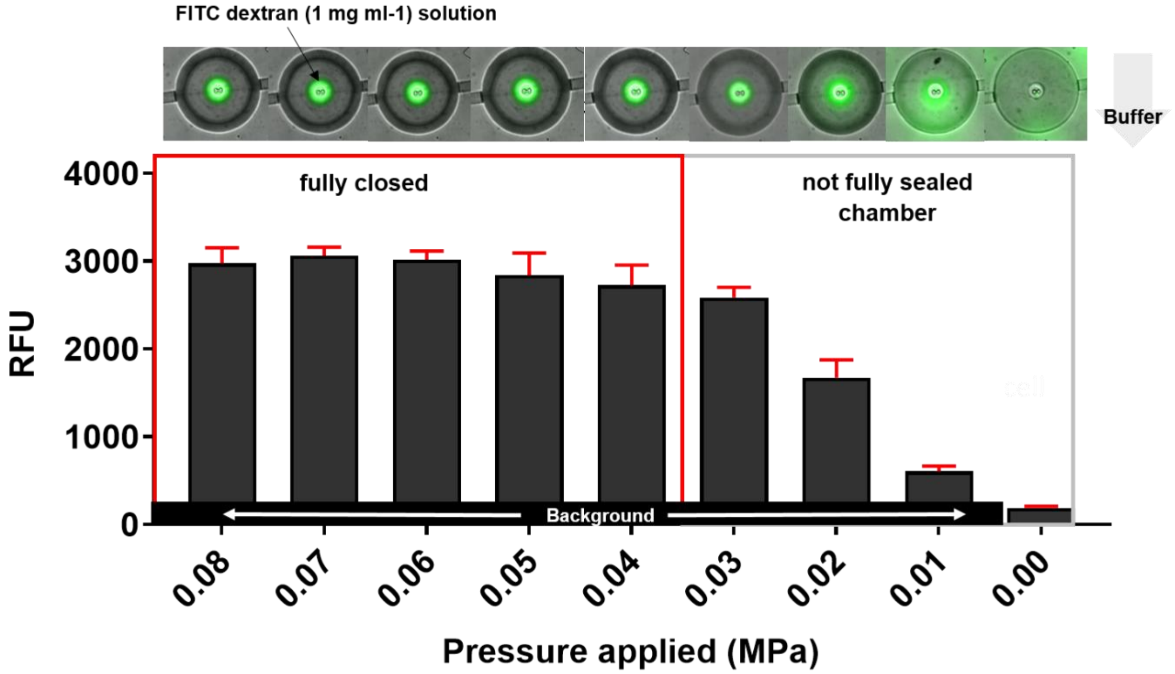

Figure S3. To create a sealed microchamber, the pneumatic valves were applied with positive pressure using an air pump (ULVAC DA-60S). The working pressure was determined by actuating the valves at different pressure and measuring the fluorescence of the compartmentalized FITC dextran solution (1 mg ml^{-1}, Sigma-Aldrich) inside and outside of the chamber. An applied pressure of 0.07 MPa was later on used in the conduct of the GrB activity assay experiment.

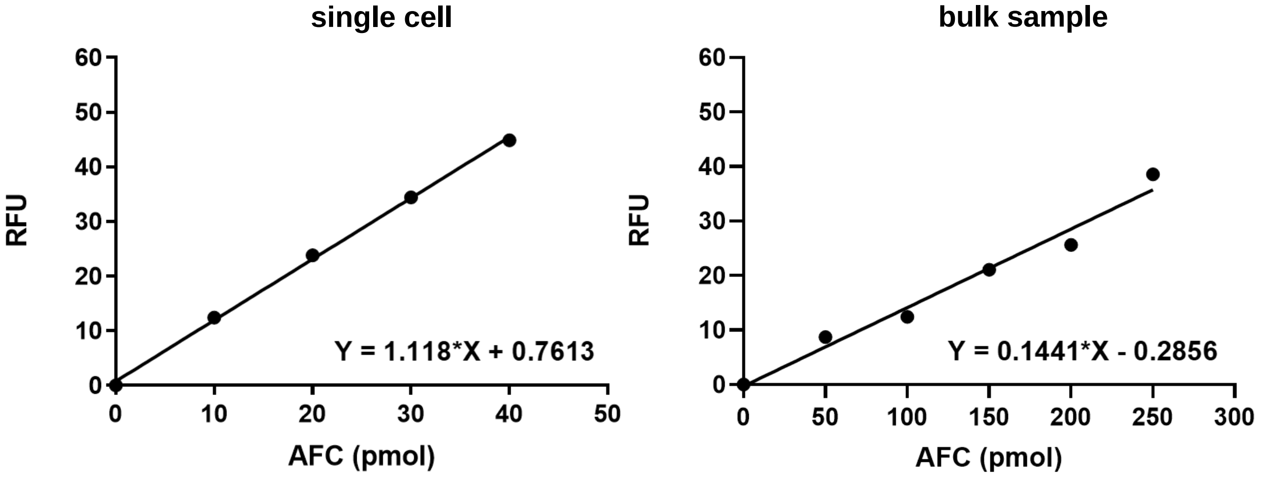

Figure S4. Standard curves were taken using the microfluidic device (left) for single cell measurement and a 96-well plate (right) for bulk sample measurement. Different amounts of AFC standard were placed in the microfluidic platform as well as in the well-plate following the assay kit protocol. The fluorescence coming from each of the sampled micro chamber and each well of the 96-well plate was measured. This was used for the interpolation of the GrB activity in the assay experiment.

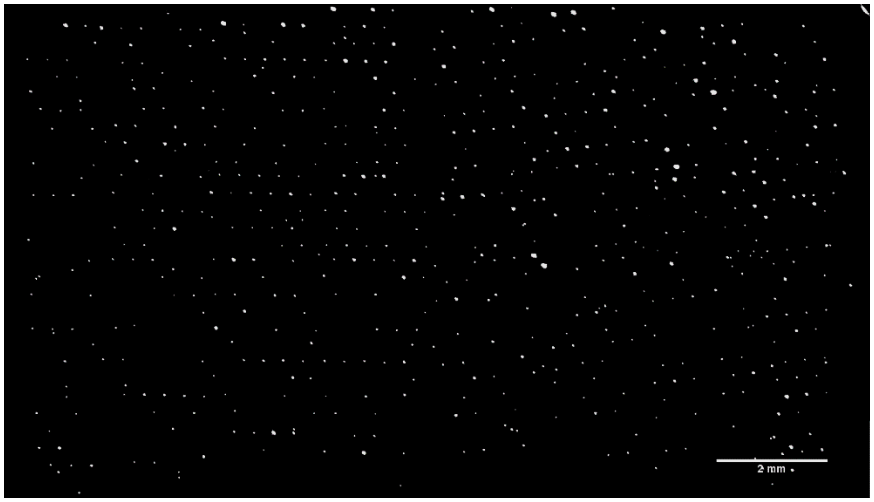

A. Fluorescent samples caught in the hydrodynamic traps at 5 µL/min flow rate

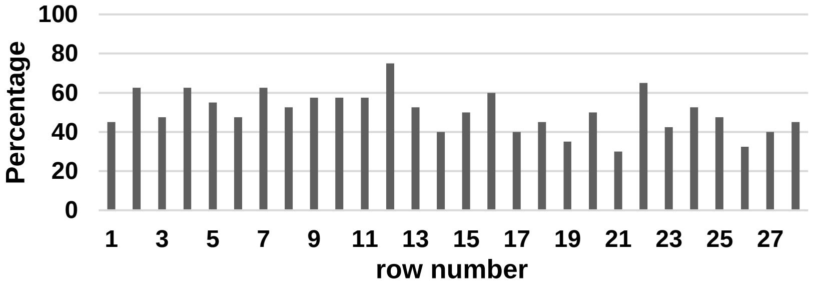

Percentage of traps occupied for 5µl/min flow rate

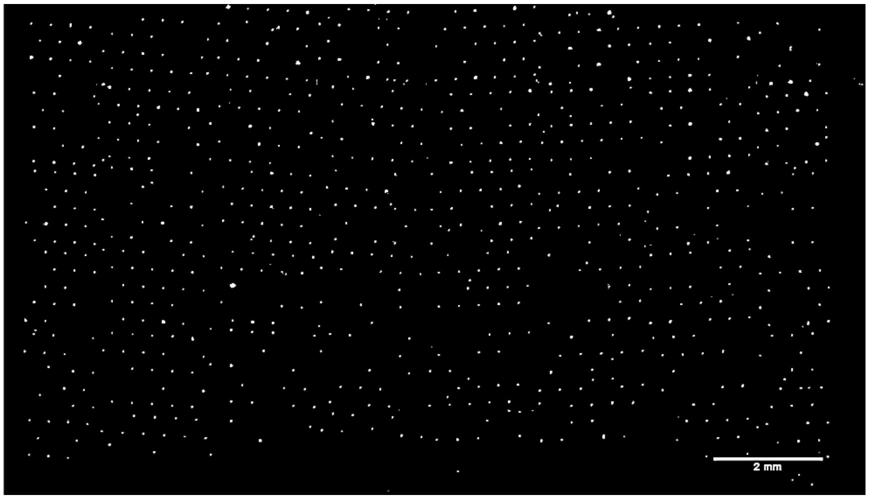

B. Fluorescent samples caught in the hydrodynamic traps at 10 µL/min flow rate

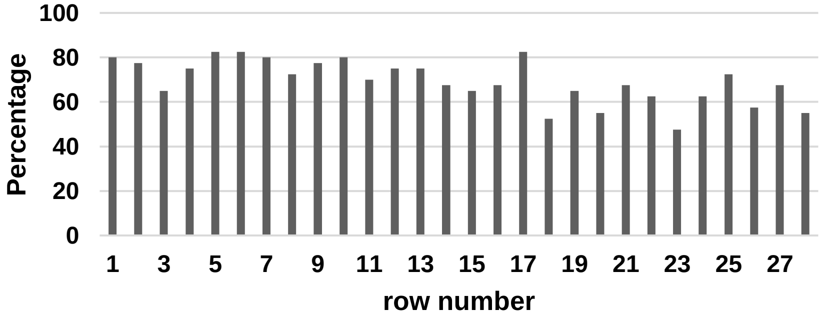

Percentage of traps occupied for 10 µl/min flow rate

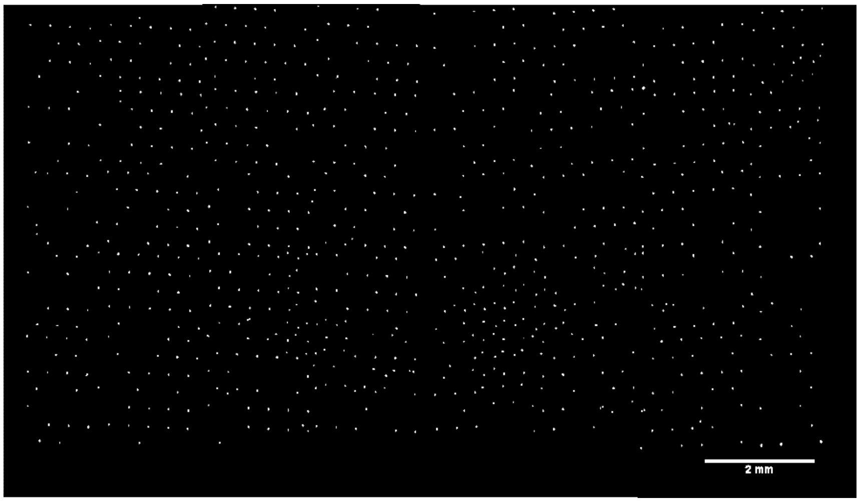

C. Fluorescent samples caught in the hydrodynamic traps at 15 µL/min flow rate

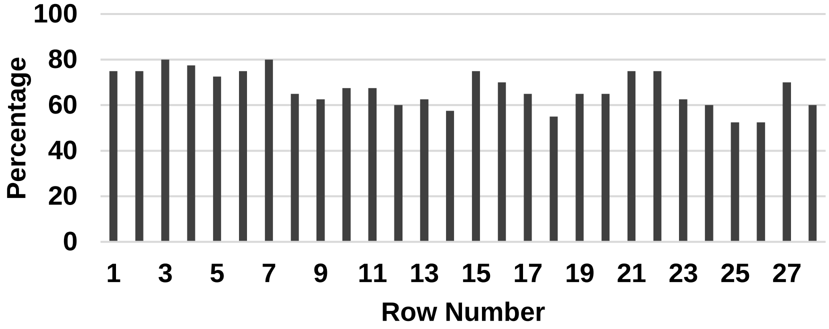

Percentage of traps occupied for 15 µl/min flow rate

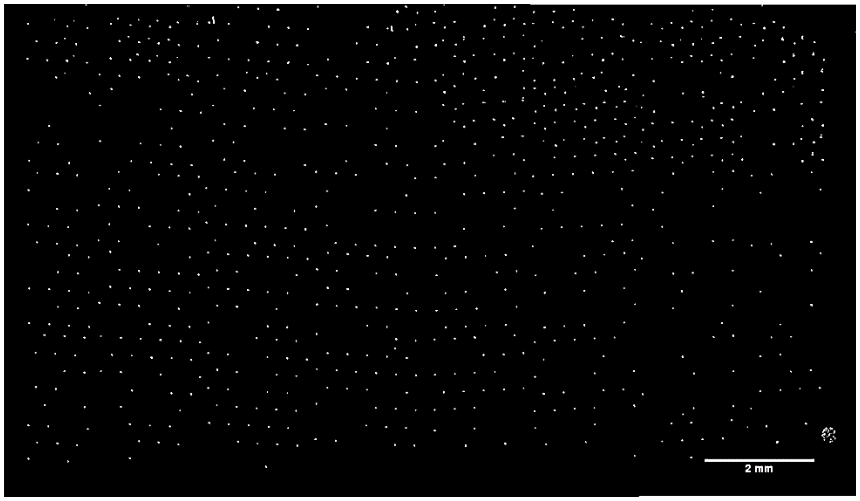

D. Fluorescent samples caught in the hydrodynamic traps at 20 uL/min flow rate

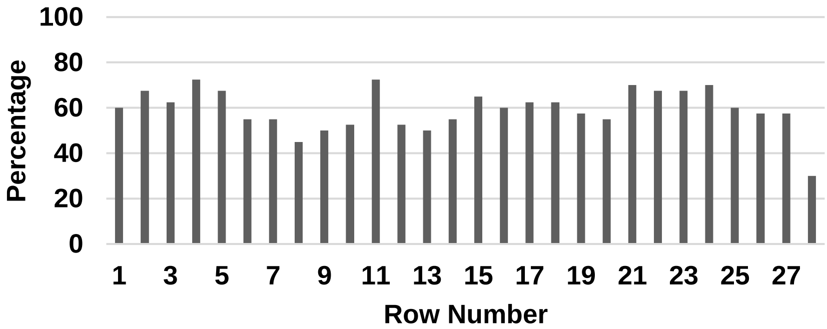

Percentage of traps Occupied for 20 µL/min Flow Rate

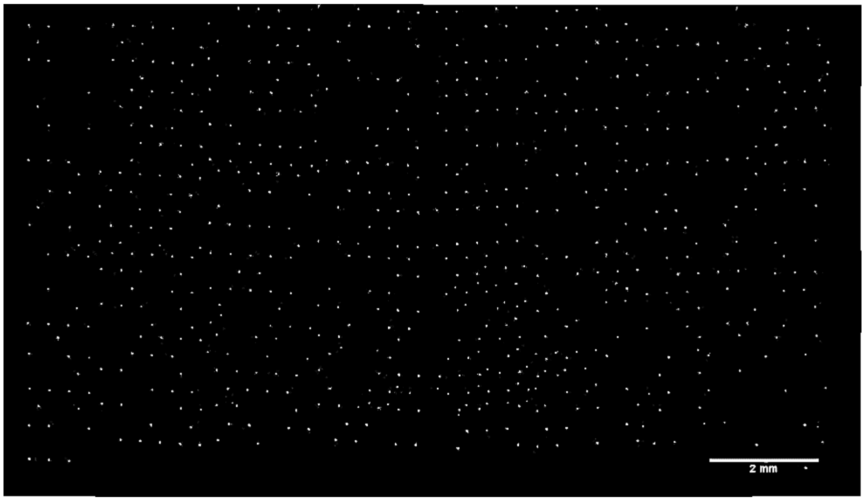

E. Fluorescent samples caught in the hydrodynamic traps at 30 µL/min flow rate

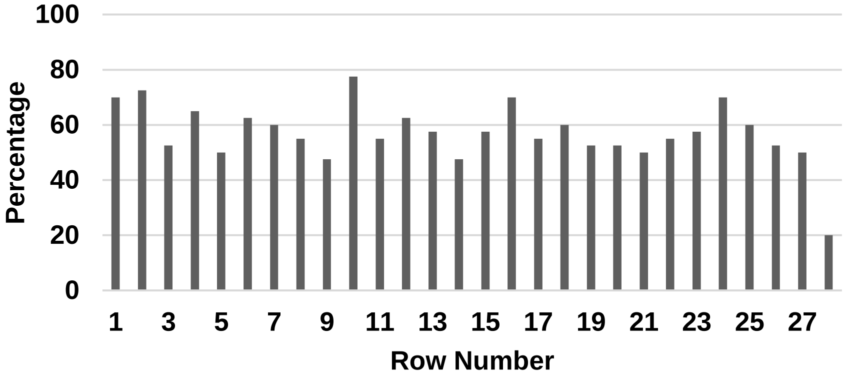

Percentage of traps Occupied for 30 µL/min flow rate

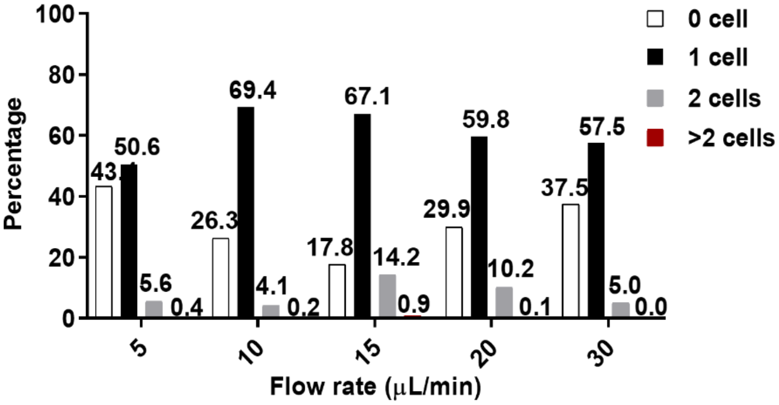

F. Percentage of trap occupancy at different flow rates. The percentage of traps with single capture is shown by the black solid bar, double capture by gray solid bar, while more than 2 cells captured is shown by red bar

Figure S5. The microfluidic device was tested for its ability to trap fluorescent cells at different flow rates. Sixty microliters (60 µL) of cell suspension (10^6 cells mL^{-1}) were made to flow at (A) 5, (B) 10, (C) 15, (D) 20, and (E) 30 µL min^{-1} flow rates. A summary of the percentages of the trap occupancy for single, double, and > 2 cells is shown in F.

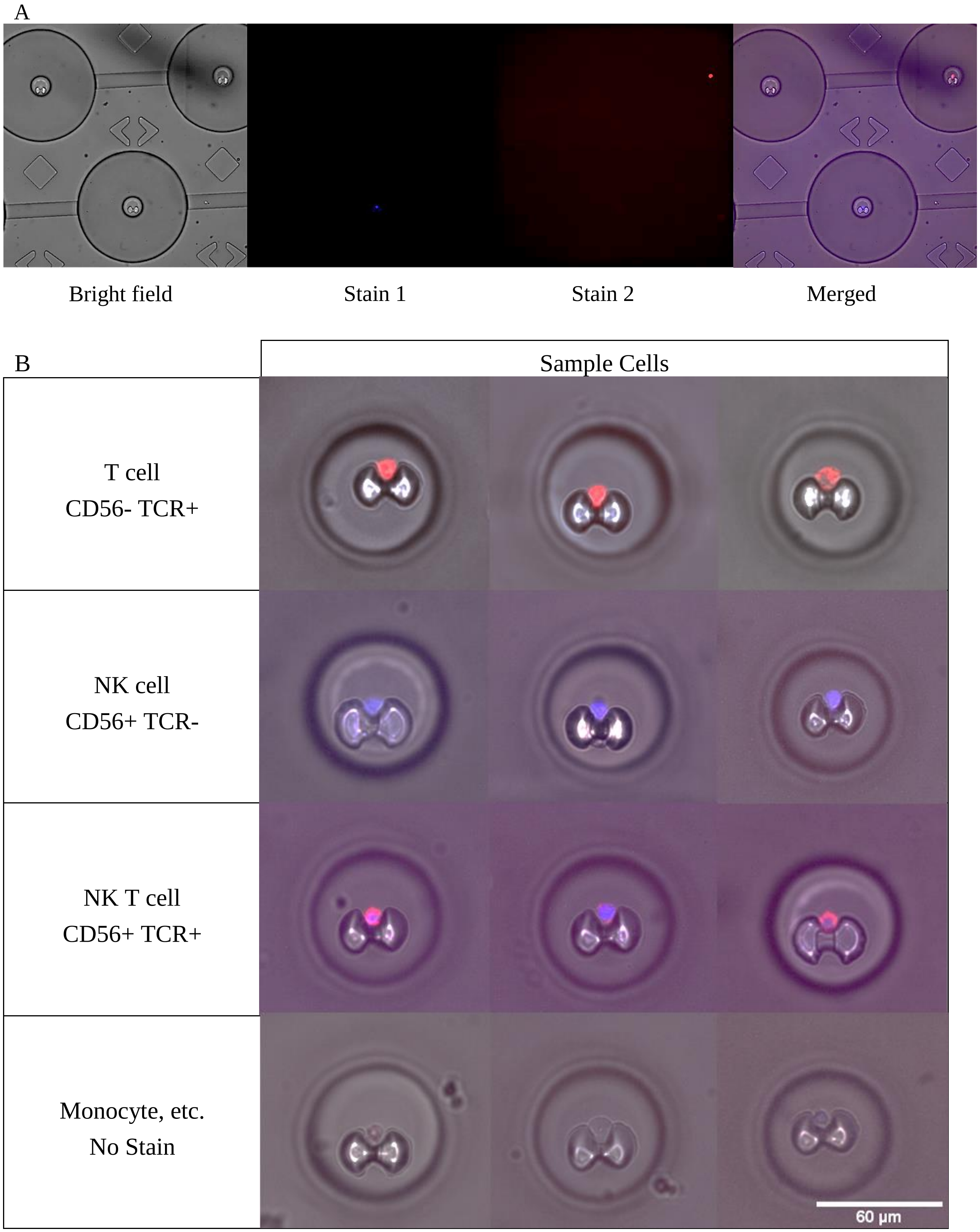

Figure S6. Cell surface marker staining was performed on-chip to determine the composition of PBMC samples. FC receptor blocking was first performed before a mixture of antibody markers were introduced into the chip. A) Large view of the same chambers indicating the multiple staining done on the cells. B) Depiction of cell identification utilizing fluorescence measurement of antibody stain.
